# Prevalence and Determinants of Never Treatment During Mass Drug Administration for Lymphatic Filariasis: A Systematic Review and Meta-Analysis

**DOI:** 10.3390/healthcare14182962

**Published:** 2026-09-10

**Authors:** Devi Das, Shukla Mandal, Uday Mondal, Mihir Bhatta, Deepanjan Ray, Anoop Velayudhan, Bobby Paul, Saibal Das, Indranil Saha

**Affiliations:** 1 Department of Epidemiology, National Institute for Research in Bacterial Infections, Indian Council of Medical Research, Kolkata 700010, West Bengal, India; devi06das@gmail.com (D.D.); optomshukla.m@gmail.com (S.M.); udaym794@gmail.com (U.M.); 2 Department of Clinical Research, National Institute for Research in Bacterial Infections, Indian Council of Medical Research, Kolkata 700010, West Bengal, India; mihirbhatta@gmail.com (M.B.); saibal.das@icmr.gov.in (S.D.); 3 Independent Researcher, Kolkata 700094, West Bengal, India; babai.bsmc@gmail.com; 4 Department of Epidemiology and Communicable Disease, Indian Council of Medical Research, New Delhi 110029, India; anoopvel@gmail.com; 5 Department of Preventive & Social Medicine, All India Institute of Hygiene & Public Health, Kolkata 700073, West Bengal, India; drbobbypaul@gmail.com; 6 Department of Biological Sciences, Academy of Scientific and Innovative Research (AcSIR), Ghaziabad 201002, Uttar Pradesh, India; 7 Department of Global Public Health, Karolinska Institutet, 171 77 Stockholm, Sweden; 8 Department of Medical Research, Academy of Scientific and Innovative Research (AcSIR), Ghaziabad 201002, Uttar Pradesh, India; 9 Model Rural Health Research Unit (MRHRU), National Institute for Research in Bacterial Infections, Indian Council of Medical Research, Darjeeling 734012, West Bengal, India

**Keywords:** lymphatic filariasis, never treated, mass drug administration, prevalence, determinants, systematic review, meta-analysis, preventive chemotherapy

## Abstract

Objectives: The term “never treated” refers to individuals who self-report having never ingested tablets during any round of Mass Drug Administration (MDA) against Lymphatic Filariasis (LF). This study estimated the pooled prevalence of never-treated individuals with MDA for LF and identified factors associated with persistent non-treatment. Methods: This systematic review and meta-analysis was registered in PROSPERO [CRD420251270067] and conducted in accordance with the PRISMA 2020 guidelines. Relevant literature was identified by searching databases, i.e., PubMed, Scopus, Cochrane Central, Embase, Web of Science, and Epistemonikos from January 2000 to December 2025, and only studies published in English were included. The AXIS tool was used to assess the study quality. A random-effects model with 95% CI was applied to estimate the pooled prevalence. Subgroup, leave-one-out sensitivity, and meta-regression analyses were conducted to explore the heterogeneity. Results: Eighteen studies including 24,940 individuals showed the pooled prevalence of never treatment during all MDA rounds at 26% (95% CI: 16% to 37%, I^2^ = 99.7%, 95% prediction interval: 0% to 75%). Exploratory subgroup analysis indicated wide regional and MDA round-specific variations. Meta-regression indicated that publication year did not explain the variability. Common reasons for persistent non-treatment included fear of side effects, limited knowledge, difficulty swallowing the pills, and absence during drug distribution. Conclusions: Persistently never-treated populations may represent an important programmatic barrier to LF elimination. Given the very low certainty of evidence, extreme heterogeneity, and potential publication bias, these estimates should be interpreted cautiously. Future research should ensure broader geographical representation, including both urban and rural areas, and explore structural, behavioural, and socio-cultural determinants to better target high-risk groups.

## 1. Introduction

Lymphatic Filariasis (LF) is a mosquito-transmitted neglected tropical disease (NTD) and is considered “eradicable” or “potentially eradicable” [1,2]. According to the World Health Organization (WHO), LF remains endemic in tropical and subtropical regions, with millions infected and over 657 million people at risk globally [3]. In Asia, LF is endemic in the Indian subcontinent, Southeast Asia, and the Pacific Islands, with India, Indonesia, and the Philippines bearing a substantial proportion of the global burden [4]. Southeast Asia, with nine endemic countries, reports about 45.5 million LF cases [4,5]. In Africa, countries including Ghana, Nigeria, and the Democratic Republic of Congo report some of the highest LF cases [6]. Together, a number of countries (India, Indonesia, Bangladesh, and Nigeria) contribute the majority of the global LF burden [3].

Although LF is rarely fatal, its progression to chronic manifestations, i.e., lymphedema of limbs, genitalia, scrotal hydrocele, and rheumatic manifestations, results in physical disability [7,8]. These conditions restrict mobility, reduce productivity, and increase dependence on their family members, leading to economic consequences [9,10]. Affected individuals often face social stigma and psychosocial impacts like anxiety, depression, distress, social impairment, and suicidal thoughts [11]. Thus, households, particularly those in low-income settings, face considerable caregiving costs [12], contributing to a decline in the quality of life [10].

To eliminate this disease, WHO launched the Global Programme to Eliminate Lymphatic Filariasis (GPELF), aiming to interrupt transmission through preventive chemotherapy via mass drug administration (MDA). The MDA programme targets all eligible people aged 2 and above with either two- or three-drug strategies (typically combinations of albendazole, ivermectin, and/or diethylcarbamazine), depending on co-endemicity with other NTDs and country-specific strategies [13,14]. Its primary prophylactic goal is to clear microfilariae from the blood, thereby halting transmission when administered at high coverage. Achieving at least 65% coverage of the total at-risk population is essential to effectively reduce microfilaria reservoirs and interrupt transmission [14]. However, high reported coverage does not necessarily reflect actual drug ingestion at the individual level.

Previous literature has demonstrated a discrepancy between the coverage (receipt of the drug) and the compliance (actual ingestion) of the drugs, with compliance often lower than reported coverage [15,16,17,18]. A particularly concerning subgroup is the “never treated” population [19,20]. The term “never treated” refers to those individuals who self-report that they have never ingested LF tablets during any round of MDA, including people who consistently remain outside the reach of the health system during drug distribution [20]. These individuals may contribute to ongoing transmission by acting as a persistent reservoir for infection, and have been shown to exhibit two-fold higher circulating filarial antigen levels [19,20,21]. The literature notes that never treatment patterns are context-specific, influenced by factors like lack of social and family support, working in formal sectors, perceived vulnerability to adverse drug reactions, difficulty recalling drug timings, absence of household drug use, and lack of knowledge [20,22,23].

To meet GPELF 2030, LF must be eliminated as a public health issue in 80% of endemic countries by identifying and treating remaining populations [24]. While previous reviews have documented irregular compliance, there is a specific lack of quantitative pooling focused exclusively on the persistently ‘never treated’ sub-population. This present systematic review and meta-analysis (SRMA) provides a comprehensive synthesis of studies to estimate the pooled prevalence of individuals never treated and to synthesize the factors associated with non-treatment across all MDA rounds.

## 2. Materials and Methods

This SRMA was prospectively registered in the PROSPERO database [CRD420251270067] to avoid research duplication and to uphold methodological rigour. The study was conducted in adherence to PRISMA-2020 guidelines to ensure reproducibility (Supplementary Table S1) [25].

Literature search: To structure the literature search, the research questions were divided into the key elements following the CoCoPop (Condition, Context, and Population) framework, with treatment adherence as the Condition (Co), mass drug administration as the Context (Co), and patients with lymphatic filariasis as the Population (Pop). Researchers associated with the Joanna Briggs Institute (JBI), most notably Munn and his colleagues, were credited in their foundational 2018 methodological work on systematic reviews of prevalence and incidence data [26]. Relevant MeSH terms for each concept were identified, and a search strategy was built using Boolean operators. “OR” included synonyms within concepts, while “AND” combined concepts for precise results. Six databases, i.e., PubMed, Scopus, Cochrane Central, Embase, Web of Science, and Epistemonikos, were searched to find our relevant literature between January 2000 and December 2025 (Supplementary Table S2). Forward (citation tracking) and backward (reference screening) searches were conducted to ensure comprehensive literature inclusion.

Screening process: All records were imported to Rayyan for deduplication after retrieval. Two independent reviewers then screened articles by title-abstract (TiAb) and full-text review, and a third reviewer resolved discrepancies. Inter-rater agreement was strong (Cohen’s kappa = 0.85).

Study selection criteria: This review included observational studies reporting the prevalence of individuals never treated during MDA for LF or identifying factors associated with this group. For this review, “never treated” (persistent non-participation) was operationally defined as individuals who self-reported that they had not ingested any dose of albendazole, ivermectin, or diethylcarbamazine during any round of MDA conducted in their respective area. The exact survey question, recall period, and metric for each study were explicitly extracted. Studies involving participants of any age and in any setting were eligible, but intervention-based designs, such as RCTs and quasi-experimental studies, were excluded. The review also excluded case reports, series, opinions, reviews, commentaries, abstracts, editorials, and studies without full text. Only studies published in English were accepted for review.

Data extraction and quality assessment: Two reviewers independently extracted data using a standardized Cochrane Data Extraction Form [27]. Data included publication year, country, duration, design, sample size, demographics, prevalence, and factors related to never-treated status. We used the AXIS tool to assess study quality because its domains were explicitly designed to appraise cross-sectional studies, the only study design included in this review [28].

Statistical analyses: The meta-analysis was conducted using R statistical software (version 4.5.2) in the RStudio integrated development environment (version 2026.01.0 + 392), using the *meta* (version 8.5-0) and *metafor* (version 5.0-1) packages and the *metaprop* function. The pooled prevalence of individuals who had never been treated with MDA was estimated using a random-effects model with restricted maximum likelihood (REML), and results were presented with 95% confidence intervals (CI). Proportions were stabilized using the Freeman–Tukey double arcsine transformation, with a 0.5 continuity correction applied where necessary. Confidence intervals were calculated using the Clopper–Pearson method, and the Hartung–Knapp–Sidik–Jonkman (HKSJ) adjustment was applied to account for uncertainty in the estimation of between-study heterogeneity. Publication bias was assessed using Begg and Mazumdar test, in addition to visual inspection of funnel plot asymmetry [29]. Subgroup analyses were conducted by number of previous rounds of MDA, and geographical categorization of study countries by WHO region to explore sources of heterogeneity [30]. Leave-one-out sensitivity analysis and Baujat plots were performed to identify influential studies. Meta-regression analysis was performed, with publication year as a covariate, to assess temporal effects on the pooled prevalence.

To assess the certainty of the evidence for each outcome across studies, a GRADE (Grading of Recommendations Assessment, Development and Evaluation) assessment was conducted by examining the risk of bias, inconsistency, indirectness, imprecision, and publication bias. A summary of findings table was generated to present the overall certainty and to guide interpretation of the results.

## 3. Results

**Figure 1:**
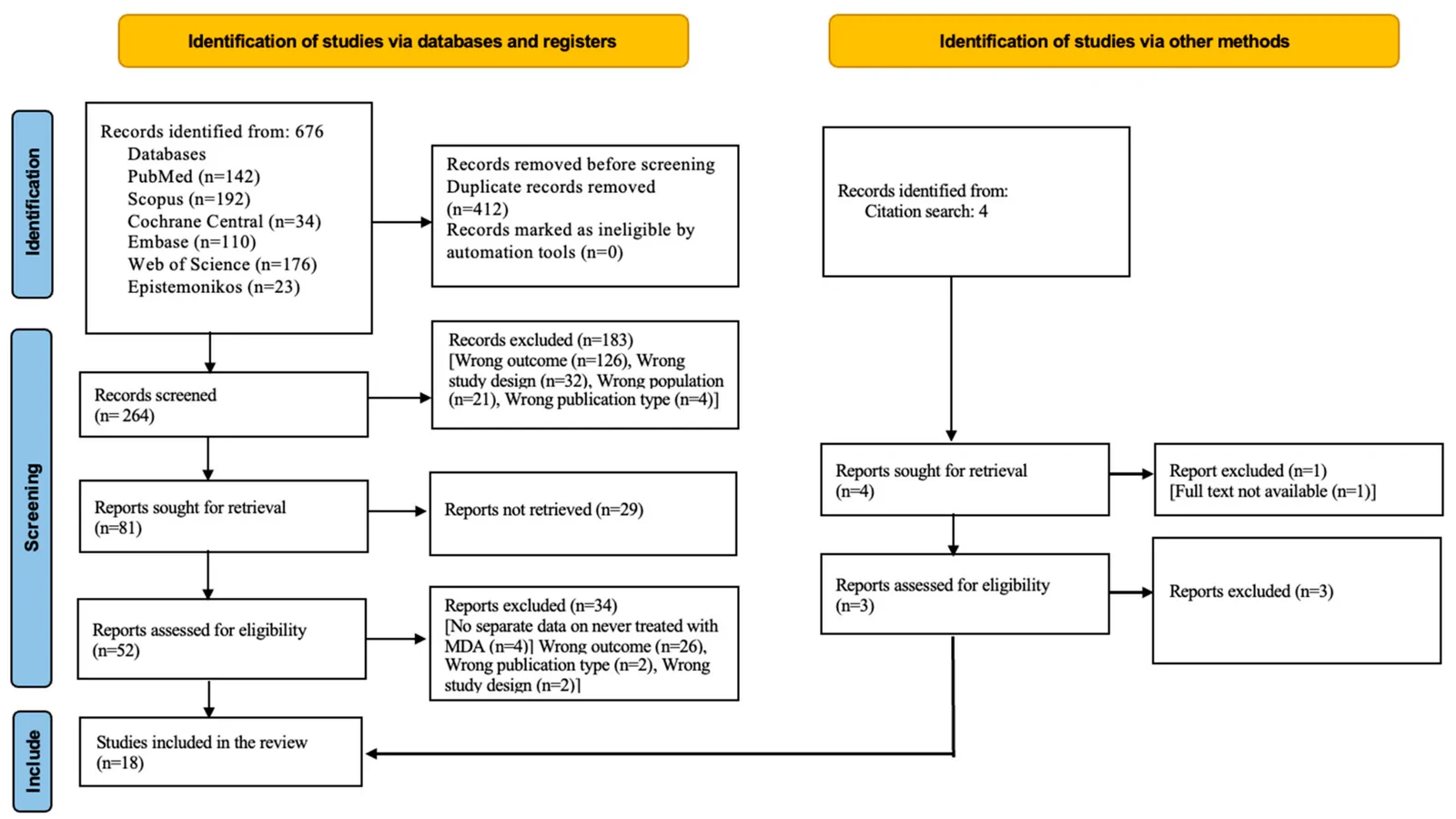
Flowchart for literature search and study selection following the PRISMA guidelines.

Six electronic databases identified 676 studies. Following two stages of screening, 18 studies met the inclusion criteria [19,21,22,23,31,32,33,34,35,36,37,38,39,40,41,42,43,44]. The PRISMA flowchart (Figure 1) presents the reasons for exclusion and the number of excluded studies.

All 18 included studies reported the prevalence of individuals who had never been treated with MDA [19,21,22,23,31,32,33,34,35,36,37,38,39,40,41,42,43,44]. Of these, six studies further addressed the factors associated with persistent non-treatment across all MDA rounds (Table 1) [19,22,23,34,36,39]. Thirteen studies explicitly reported the study period, spanning from 2007 to 2024. The geographical distribution of studies comprising American Samoa (*n* = 3) [41,42,44], Guyana (*n* = 2) [23,39], Haiti (*n* = 2) [22,33], India (*n* = 2) [36,38], Tanzania (*n* = 2) [21,35], Egypt (*n* = 1) [40], Ghana (*n* = 1) [32], Indonesia (*n* = 1) [19], Myanmar (*n* = 1) [34], Nepal (*n* = 1) [37], Samoa (*n* = 1) [43], in addition to one study conducted across multiple countries [31] (Table 1). By WHO region, five studies were from the Western Pacific region, four each from the Americas and Southeast Asia, three from the African region, and one from the Eastern Mediterranean region. All included studies used a community-based cross-sectional design, except one conducted in a workplace setting. Sample sizes ranged widely from 101 to 4420. The study populations covered a broad age range (2 to 95 years). Most studies targeted adolescent and adult populations, while a limited number included younger children or older adults. Most studies included both males and females, with female participation slightly higher in some studies (Table 1).

Quality assessment with AXIS showed 100% of studies clearly defined aims and used suitable designs. Half justified their sample size, while 94.44% used piloted or published instruments. Results were consistent, basic data well described, and ethical approval or consent obtained in all studies (Supplementary Table S3).

Publication bias and small-study effects were assessed using the Begg and Mazumdar test, only for comparisons involving 10 or more studies. In accordance with GRADE recommendations for proportion meta-analyses, funnel plot asymmetry was interpreted cautiously as reflecting small-study effects, methodological differences, or clinical heterogeneity rather than definitive evidence of unpublished literature.

**Figure 2:**
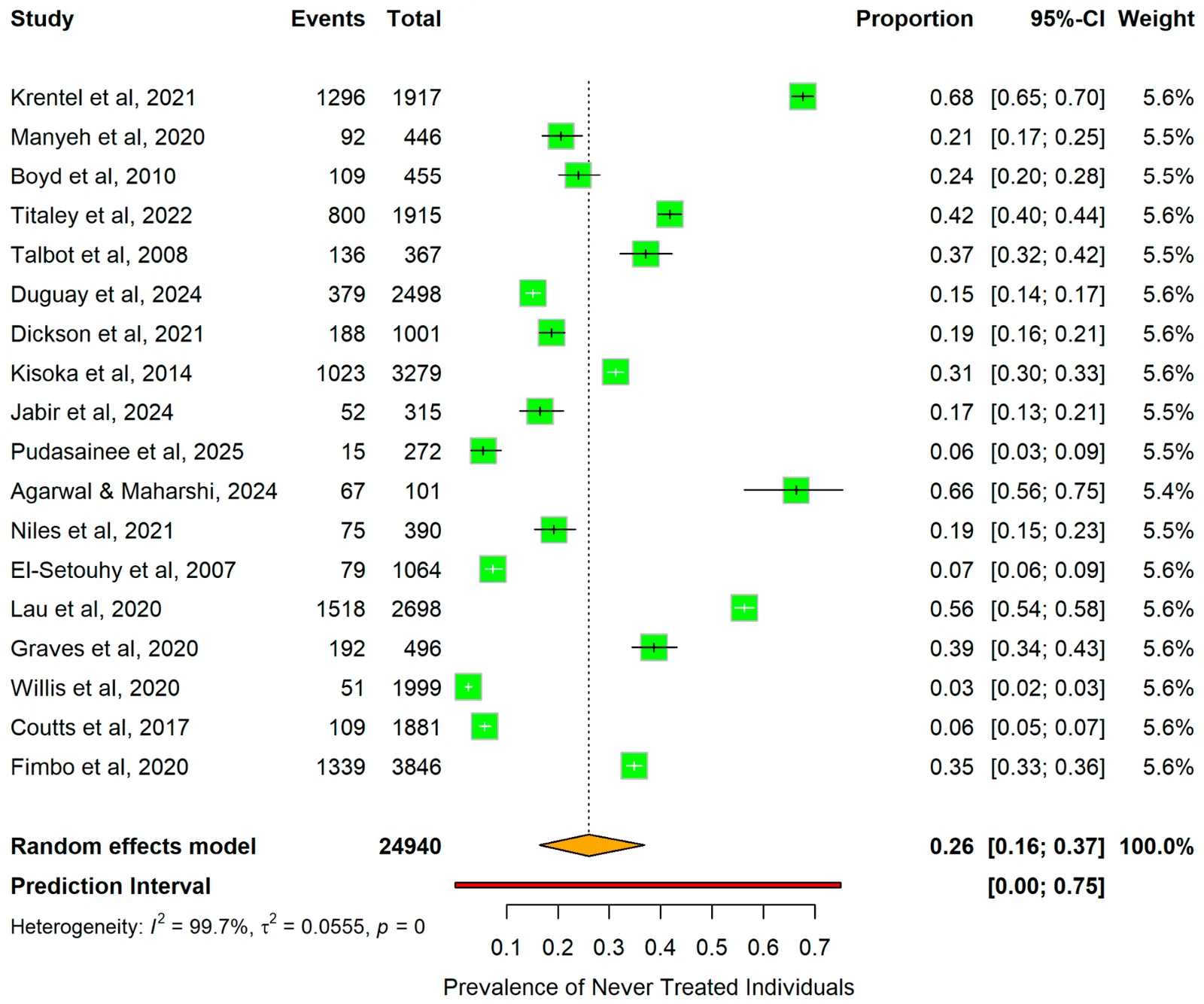
Forest plot showing the pooled prevalence of individuals who were never treated with MDA across 18 studies [19,21,22,23,31,32,33,34,35,36,37,38,39,40,41,42,43,44]. The model incorporates a Freeman–Tukey double arcsine transformation and a prediction interval to account for substantial between-study heterogeneity.

The prevalence of individuals who had never been treated with MDA ranged from 2.55% to 66.34%. A meta-analysis of 18 studies involving 24,940 individuals estimated a pooled prevalence of 26% (95% CI: 16% to 37%, 95% prediction interval: 0% to 75%) with an I^2^ value of 99.7% (Figure 2). Visual inspection of the funnel plot suggested asymmetry, and Begg and Mazumdar’s rank correlation test (*p* < 0.05) indicated potential small-study effects or reporting asymmetry (Supplementary Figure S1). When categorized by WHO region, the pooled prevalence of those never treated was highest in the Western Pacific region (60%, 95% CI: 10% to 95%), followed by the South-east Asia region (33%, 95% CI: 9% to 72%) and the region of Africa (28%, 95% CI: 22% to 35%) (Supplementary Figure S2). The number of previous MDA rounds varied across study settings, ranging from no prior MDA to >10 rounds, with subgroup analysis showing prevalence estimates of 100% for no MDA (95% CI: 99% to 100%), 27% (95% CI: 19% to 36%) for ≤5 rounds, 21% (95% CI:11% to 37%) for 6–10 rounds, and 19% (95% CI: 6% to 46%) for >10 rounds (Supplementary Figure S2).

The leave-one-out sensitivity analysis demonstrated that exclusion of any single study did not substantially alter the overall pooled prevalence estimate, which ranged from 21% to 26%, and heterogeneity remained consistently high (I^2^ ≈ 99%) (Supplementary Figure S3). The Baujat plot identified three studies, i.e., Lau et al., 2020, Duguay et al., 2024, and Coutts et al., 2017, as contributing to heterogeneity, suggesting potential outlier effects (Supplementary Figure S4) [23,41,44].

**Figure 3:**
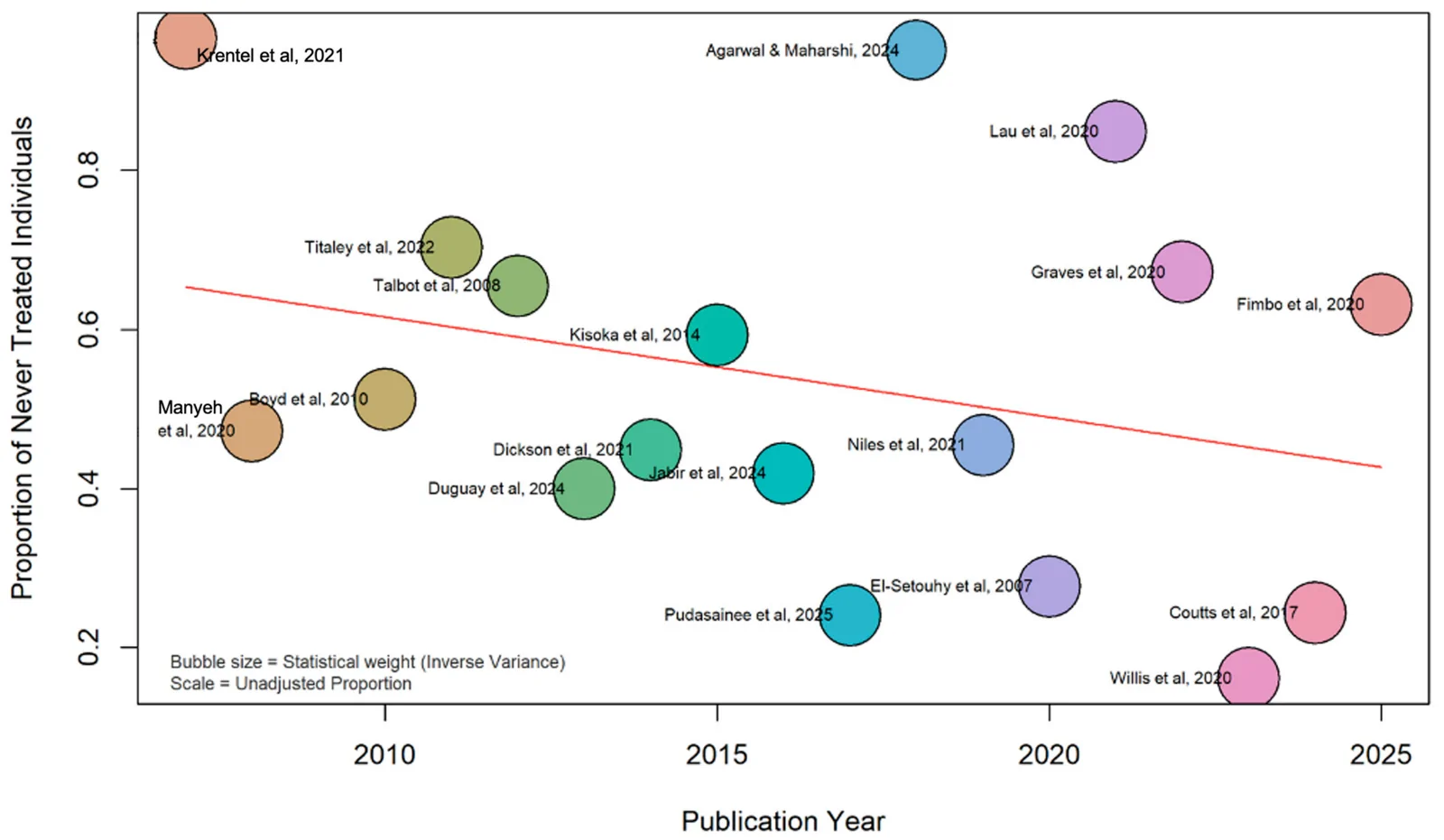
Bubble plot of meta-regression assessing the time trend in the prevalence of individuals who were never treated with MDA (*n* = 18) [19,21,22,23,31,32,33,34,35,36,37,38,39,40,41,42,43,44]. Bubble size is proportional to the statistical weight of each study within the random-effects model (inverse variance). The y-axis displays the unadjusted raw proportion of never-treated individuals, while the fitted meta-regression line represents the relationship across publication years.

A mixed-effects meta-regression was performed to investigate possible temporal trends, incorporating publication year as a covariate (k = 18). The analysis showed no significant association between publication year and the pooled proportion (QM(1) = 0.0058, *p* = 0.9394). The regression coefficient was negligible (β = −0.0046, 95% CI: −0.12 to 0.11), and the model explained none of the observed heterogeneity (R^2^ = 0.00). Significant residual heterogeneity persisted (QE(16) = 2773.24, *p* < 0.0001, I^2^ = 99.73%), indicating that the variance in prevalence estimates was driven by factors other than the chronological timing of the research. As illustrated in the bubble plot, the regression line remained relatively horizontal (Figure 3).

**Figure 4:**
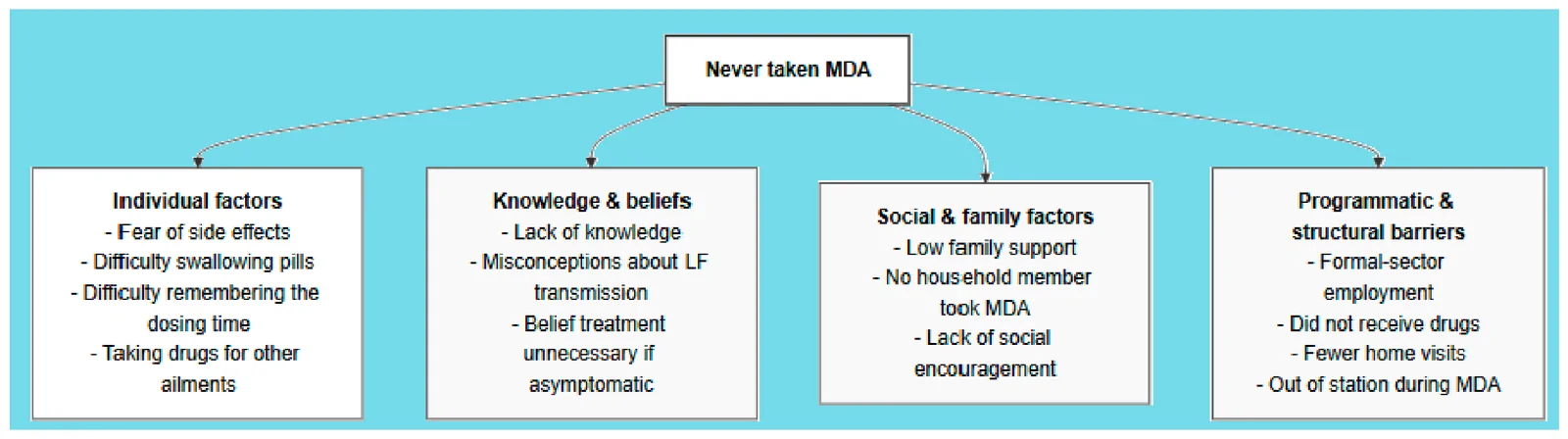
Thematic framework diagram showing factors associated with persistently never taking MDA (*n* = 6) [19,22,23,34,36,39]. *Note on Qualitative Synthesis:* To construct this framework, reported reasons and statistically significant determinants for non-treatment were systematically extracted from the included studies. These factors were then qualitatively coded and iteratively grouped into broader conceptual domains based on shared contextual characteristics. This synthesis resulted in four distinct domains: Individual factors, Knowledge and beliefs, Social and family factors, and Programmatic and structural barriers.

Six studies reported that never taking MDA for LF is influenced by a combination of individual, social, and programmatic factors (Figure 4). Common reasons included fear of side effects and perceived vulnerability to adverse events. Limited knowledge about LF, poor understanding of its transmission, and low awareness of the importance of preventive treatment contributed to never taking MDA. Other barriers include difficulty swallowing pills, uncertainty about taking full doses, forgetting the scheduled time, being absent when the drug was distributed, or not receiving the drugs. Social influences are important, as individuals with low family or community support, or whose family members did not take the drug, were less likely to participate.

GRADE Analysis: The present review provided very low certainty that a substantial proportion of individuals remain never treated during MDA programmes. Outcomes from the subgroup analysis based on regional variation, and declining prevalence with increasing MDA rounds also demonstrated very low certainty, primarily due to serious risk of bias, inconsistency, imprecision, and publication bias. The narrative outcome regarding the common reasons for never taking MDA was likewise rated as low certainty (Supplementary Figure S5).

## 4. Discussion

This SRMA provides the first pooled estimate of the prevalence of individuals who had never been treated with MDA for LF. Across 18 included studies, with 24,940 individuals, 26% reported that they had never ingested any of the three anti-filarial drugs during any MDA round. The presence of individuals who have never been treated is a significant concern from both clinical and epidemiological perspectives, regardless of the underlying reasons for non-treatment. Infected individuals of this group remain at a higher risk of clinical deterioration and act as reservoirs for disease transmission [20]. Previous literature from multiple settings highlights the persistent gaps in MDA uptake despite repeated implementation cycles. A study from Guyana reported that 19.23% of individuals had never ingested MDA, regardless of whether the programme utilized dual or triple drug regimens [39]. This finding aligns with other studies from other settings. For instance, studies from India and Nepal reported that 16.51% and 5.51% were never treated after 18 and 14 rounds of MDA, respectively [36,37]. Several studies have also shown an association between MDA participation and infection status. In Samoa, antigen prevalence was higher among individuals who had never received MDA than among those who had participated in at least one MDA round [43]. Conversely, a study in American Samoa showed that ever taking MDA was significantly protective (*p* = 0.04) against infection compared to non-treatment [44]. The GPELF aims to interrupt transmission by reducing microfilariae prevalence below 1% through at least five rounds of MDA [45]. Mathematical transmission models such as LYMFASIM and TRANSFIL provide strong evidence that systematic non-adherence (i.e., never-treated individuals) can sustain transmission in high-endemicity settings [1,20]. Despite the WHO 2030 elimination target, persistence of this cohort threatens programme success and may prolong the elimination timeline [45].

Regional subgroup analysis based on WHO criteria revealed variability in the pooled prevalence of individuals who were never treated. The highest prevalence was observed in the Western Pacific region (60%), followed by the South-east Asia region (33%), and the region of Africa (28%). Of the 47 countries within the WHO African region, 31 countries require preventive chemotherapy for LF [46]. Progress in MDA distribution in some countries in this region is hindered by delayed geographical mapping of LF, largely due to limited access and political instability [46]. Comprehensive mapping remains a prerequisite for the effective implementation of any targeted public health intervention [47]. In the Americas, high-coverage MDA using triple-drug therapy may accelerate LF elimination. However, persistent gaps in morbidity management and the underestimation of disease burden remain significant barriers to achieving comprehensive programme goals [48].

Subgroup analysis by number of prior MDA rounds revealed a declining trend in never-treated prevalence from 100% with no MDA to 19% after more than 10 rounds, suggesting that repeated rounds progressively reduce non-participation, though the persistence of a substantial never-treated proportion (19%) even beyond 10 rounds indicates that repeated implementation alone is insufficient to eliminate this group. Participation appears to depend on a complex interplay of factors, including community trust in government and healthcare workers, individual knowledge of LF, prior experience with adverse events, and social influence [49].

Sex-specific subgroup analysis was not performed in our study because the available sex-stratified data were insufficiently comparable across studies to support a valid pooled estimate. Nevertheless, sex-based differences in MDA participation have been reported in individual studies across different settings. A multi-country analysis showed a higher proportion of never treatment among males (M: 43.29%; F: 41.25%), findings also observed in Indonesia (M: 50.39%; F: 37.42%) and Guyana (M: 21.7%; F: 18.31%) [19,31,39]. Higher prevalence among males is attributed to their absence from home during the drug distribution hours due to formal employment [19]. Additionally, males have reported significantly lower knowledge of LF transmission and its asymptomatic nature [21,31]. Consequently, several studies have reported higher levels of circulating filarial antigenemia among males (8.3% and 36.2%) than among females (3.3% and 18.2%), respectively [21,31]. Although this pattern is not universal, one study reported a higher prevalence of never-treated individuals among females (65.44% vs. 50.54%), suggesting that sex-based disparities in MDA participation may be influenced by socio-cultural factors [22].

In this SRMA, only six studies examined factors associated with never treatment. The studies across diverse geographical contexts highlight that never treatment is driven by a multifaceted intersection of socio-cultural constructs and operational deficiencies. Data indicated that perceived vulnerability to adverse events and mistrust of drug safety remain primary psychological associates. For instance, in Ambon city, individuals fearing side effects have higher odds of never receiving treatment (aOR = 2.86; 95% CI: 1.99 to 4.12; *p* < 0.001) [19]. This is often supported by a lack of understanding of LF’s asymptomatic nature and the rationale for MDA in healthy individuals [39]. Social and familial support also play an important role, as evidenced by the high degree of household clustering in the case of never treatment. In one study, lack of family participation was the strongest predictor of individual non-participation (aOR = 3.93; 95% CI: 2.39 to 6.49; *p* < 0.001) [19]. Additionally, difficulty swallowing multiple MDA drugs (aOR = 3.12; 95% CI: 1.84 to 5.31; *p* < 0.001) creates a significant uptake hurdle [19]. In a specific sub-population, i.e., older adults, co-morbidities, and low trust in drug safety influenced the uptake of the MDA drugs [36]. Operational failures, i.e., inadequate frequency of household visits, high population mobility, and a “don’t know” response in surveys, indicate potential gaps in current efforts [19,39].

Future studies must move beyond measuring single-round coverage and compliance and instead prioritize identifying and characterizing the never-treated population, particularly in areas with persistent transmission [19]. Future studies should adopt standardized, validated survey instruments to operationalize and measure the “never treated” construct [20]. Never treatment data should be broken down by age, sex, and demographics to improve MDA strategies and understand why some do not participate. Understanding social factors behind never treatment is key to designing targeted interventions to boost drug uptake. Address fears of side effects, misconceptions, and lack of knowledge about LF with culturally tailored education involving trusted local figures and religious leaders. To further strengthen intervention design and implementation strategies, the RANAS framework may be applied to facilitate structured evaluation of perceived risk, social norms, ability-related barriers, and self-regulation factors, thereby guiding targeted behavioural interventions that directly address the underlying determinants of systematic non-participation [19]. Strengthening training, supportive supervision, and appropriate compensation for community drug distributors (CDDs) can improve the effectiveness of MDA programmes [50]. CDDs build trust, address misconceptions, promote participation, and ensure observed treatment, boosting uptake and reducing non-participation. Using flexible drug distribution, engaging schools and youth, and reaching migrants are key to improving accessibility and uptake. Researchers should ensure that the hard-to-reach individuals missed by MDA are not also missed by the surveys [20]. Geographical mapping of transmission hotspots and clusters of never-treated individuals is required for tailored interventions [20,46]. In urban areas, challenges like gated communities, recipient unavailability during the day, low trust in health workers, and limited awareness efforts must be systematically addressed to improve drug coverage and compliance, boost community engagement, and ensure fair access to MDA [18]. Integrating MDA distribution with other routine community health initiatives may increase acceptability and participation.

## 5. Limitation

The present study has some limitations. Only studies published in English were included in the review. Excluding literature published in other languages, unavailable literature, and grey literature may have affected the validity and generalizability of the findings. Publication bias may have influenced the pooled prevalence estimates. Substantial heterogeneity was observed across studies and persisted throughout, even in leave-one-out analysis. This heterogeneity is attributed to differences in age distribution, gender composition, study settings, survey instruments, sampling strategies, and sample sizes. Meta-regression analysis showed no significant temporal trends across publication years, suggesting that other unmeasured contextual or methodological factors may explain the observed variability. The relatively small number of studies, particularly within subgroup analysis, limits the ability to fully explore the sources of heterogeneity. Geographical representation from only 11 countries in the analysis may restrict the applicability of the findings. In addition, because the certainty of evidence remained very low across outcomes, confidence in the pooled prevalence is limited, restricting the strength of the conclusion and the generalizability of the findings.

Furthermore, recall bias critically threatens the validity of ‘never treated’ self-reports, especially in regions that have conducted over 10 historical MDA rounds. Reliance on cross-sectional survey designs also limits causal inference regarding non-treatment determinants.

The funnel plot for overall non-treatment prevalence showed visual asymmetry. However, in descriptive prevalence meta-analyses, where traditional publication filters against non-significant findings are less pronounced, this pattern is best interpreted as small-study effects driven by methodological diversity and geographic variation across survey sites rather than true publication bias.

## 6. Conclusions

Persistently never-treated populations represent an important pragmatic concern for the elimination of LF. This SRMA demonstrates that a substantial proportion of individuals remain never treated across diverse settings, although the very low certainty of evidence and substantial heterogeneity warrant cautious interpretation of the pooled estimates. Future studies should ensure broader representation from diverse geographical settings, including both urban and rural populations, and employ adequately powered sample sizes. In addition to structural determinants, exploration of behavioural and socio-cultural drivers of persistent non-treatment is required to identify and engage this high-risk group. Greater consistency in the use of standardized definitions, reporting of the population used to calculate estimates, and standardized approaches to data collection are also needed to strengthen the evidence base. Addressing systematic non-participation may help inform strategies to reach individuals who remain untreated and support progress toward eliminating LF as a public health problem.

## Figures and Tables

**Table 1:** Characteristics of included studies, prevalence of never-treated individuals with MDA, and reported factors influencing reasons for never having taken MDA (*n* = 18).

Study (Author, Year)	Country (Study Area)	Study Design	Age Group	MDA Regimen & Prior Rounds	Outcome Definition	Denominator (*n*)	Numerator (*n*)	Prevalence (%)	Determinants/Reasons for Never Taking MDA
Krentel et al., 2021 [31]	Multi-country (Fiji, Haiti, India, Indonesia, PNG)	Community-based cross-sectional	≥14 years	Variable (0 to >10 rounds depending on country)	Self-reported lifetime non-ingestion of MDA drugs	1919	812	42.30%	Not reported.
Manyeh et al., 2020 [32]	Ghana (Bole & Central Gonja)	Community-based cross-sectional	Not specified	15–18 years of rounds (Initiated 2001)	Self-reported non-ingestion across all offered rounds	446	92	20.60%	Not reported.
Boyd et al., 2010 [33]	Haiti (Leogane)	Community-based cross-sectional	3–95 years	7 rounds	Self-reported non-ingestion across all offered rounds	455	109	24.00%	Not reported.
Titaley et al., 2022 [19]	Indonesia (Ambon City)	Community-based cross-sectional	Any age group	5+ rounds (Initiated 2009, 5 add’l from 2015)	Self-reported non-ingestion of LF drugs during any round	1915	800	41.80%	Difficulty swallowing drugs, lack of family support, formal-sector employment, perceived risk of adverse events, forgot dosing time.
Talbot et al., 2008 [22]	Haiti (Leogane)	Community-based cross-sectional	≥14 years	4 rounds	Self-reported non-ingestion across all 4 rounds	367	136	37.06%	Unable/uncertain about swallowing full pill dose, lack of knowledge.
Duguay et al., 2024 [23]	Guyana (Six endemic regions)	Community-based cross-sectional	≥20 years	IDA regimen (2 rounds), prior DA since 2003	Self-reported non-ingestion of IDA/DA across all rounds	2498	379	15.20%	Lack of personal concern/belief, poor LF understanding, low acceptability & perceived importance of MDA.
Dickson et al., 2021 [34]	Myanmar (Mandalay)	Community-based cross-sectional	Not specified	6 rounds	Self-reported non-ingestion across all 6 rounds	1001	188	18.80%	Male gender, declined offered medication, lack of knowledge, fewer home visits by MDA team, young & old age groups.
Kisoka et al., 2014 [35]	Tanzania (Lindi & Morogoro)	Community-based cross-sectional	≥15 years	2 to 4 rounds (depending on specific region)	Self-reported non-ingestion across regional rounds	3279	1023	31.20%	Not reported.
Jabir et al., 2024 [36]	India (Karnataka)	Community-based cross-sectional	≥60 years	DA (15 rounds) & IDA (3 rounds)	Self-reported non-ingestion across all 18 rounds	315	52	16.50%	Fear of side effects, taking drugs for other ailments, did not receive drugs, not interested, out of station.
Pudasainee et al., 2025 [37]	Nepal (Baglung Municipality)	Community-based cross-sectional	19–78 years	14 rounds (completed by 2023)	Self-reported non-ingestion across all 14 rounds	272	15	5.50%	Not reported.
Agarwal & Maharshi, 2024 [38]	India (Jharkhand)	Community-based cross-sectional	~32.2 years (mean)	Launched 2004 (recent session 4 mos prior)	Self-reported non-ingestion across all historical rounds	101	67	66.34%	Not reported.
Niles et al., 2021 [39]	Guyana (Sentinel sites)	Community-based cross-sectional	18–89 years	Transitioning from DA to IDA therapy	Self-reported non-ingestion of any LF drugs	390	75	19.20%	No knowledge of LF, lack of awareness of transmission, lack of personal concern.
El-Setouhy et al., 2007 [40]	Egypt (Giza Governorate)	Community-based cross-sectional	≥5 years	DEC + Albendazole (5 rounds)	Self-reported non-ingestion across all 5 rounds	1064	79	7.40%	Not reported.
Lau et al., 2020 [41]	American Samoa	Community-based household & school	≥8 years	7 rounds (2000–2006)	Self-reported non-ingestion across all 7 rounds	2698	1518	56.30%	Not reported.
Graves et al., 2020 [42]	American Samoa (Worksites)	Cross-sectional Worksite survey	≥15 years	7 rounds (2000–2006)	Self-reported non-ingestion across all 7 rounds	496	192	38.71%	Not reported.
Willis et al., 2020 [43]	Samoa (Four regions)	Community-based cross-sectional	≥10 years	10 rounds pre-1999, 8 PacELF, 2 NWU	Self-reported non-ingestion across all historical rounds	1999	51	2.55%	Not reported.
Coutts et al., 2017 [44]	American Samoa	Community-based cross-sectional	≥2 years	7 rounds (2000–2006)	Self-reported non-ingestion across all 7 rounds	1881	109	5.80%	Not reported.
Fimbo et al., 2020 [21]	Tanzania (Tanga region)	Community-based cross-sectional	≥5 years	Ivermectin + Albendazole (16 rounds since 2002)	Self-reported non-ingestion across all 16 rounds	3846	1339	34.80%	Not reported.

MDA: Mass drug administration; LF: lymphatic filariasis; IDA: Ivermectin-Diethylcarbamazine-Albendazole; DA: Diethylcarbamazine-Albendazole; DEC: Diethylcarbamazine.

## Data Availability

The raw data supporting the conclusions of this article will be made available upon request.

## Supplementary Materials

The following supporting information can be downloaded at: https://www.mdpi.com/article/10.3390/healthcare14182962/s1, Figure S1: Funnel plot asymmetry showing the publication bias (*n* = 18); Figure S2: Subgroup analysis showing the pooled prevalence of individuals who were never treated with MDA based on WHO regions and the number of previous MDA rounds; Figure S3: Leave-one-out sensitivity analysis showing the stability of the pooled estimate; Figure S4: Baujat Plot showing the studies that influenced the overall pooled estimates; Figure S5: GRADE analysis was conducted based on the key quantitative outcomes and a narrative outcome; Table S1: PRISMA guideline; Table S2: Search strategy and number of retrieved articles from different databases (*n* = 6); Table S3: Quality Assessment of the selected articles by AXIS Tool for Observational Studies (*n* = 18).
