# Supplementary material for: Prevalence and Determinants of Never Treatment During Mass Drug Administration for Lymphatic Filariasis: A Systematic Review and Meta-Analysis
Source: Healthcare (Basel). 2026 Sep 10;14(18):2962. doi: 10.3390/healthcare14182962 (PMC13606423; doi:10.3390/healthcare14182962)
Supplement: Supplementary file 1 [file healthcare-14-02962-s001.zip › healthcare-4457895-supplementary.pdf]

Supplementary Table S1 PRISMA guideline

Section and Topic	Item #	Checklist item	Location where item is reported
TITLE			
Title	1	Identify the report as a systematic review.	Page 1
ABSTRACT			
Abstract	2	See the PRISMA 2020 for Abstracts checklist.	Page 1
INTRODUCTION			
Rationale	3	Describe the rationale for the review in the context of existing knowledge.	Page 2-Page 3
Objectives	4	Provide an explicit statement of the objective(s) or question(s) the review addresses.	Page 3
METHODS			
Eligibility criteria	5	Specify the inclusion and exclusion criteria for the review and how studies were grouped for the syntheses.	Page 3
Information sources	6	Specify all databases, registers, websites, organisations, reference lists and other sources searched or consulted to identify studies. Specify the date when each source was last searched or consulted.	Page 3
Search strategy	7	Present the full search strategies for all databases, registers and websites, including any filters and limits used.	Page 13-Page 14
Selection process	8	Specify the methods used to decide whether a study met the inclusion criteria of the review, including how many reviewers screened each record and each report retrieved, whether they worked independently, and if applicable, details of automation tools used in the process.	Page 3
Data collection process	9	Specify the methods used to collect data from reports, including how many reviewers collected data from each report, whether they worked independently, any processes for obtaining or confirming data from study investigators, and if applicable, details of automation tools used in the process.	Page 3-Page 4
Data items	10a	List and define all outcomes for which data were sought. Specify whether all results that were compatible with each outcome domain in each study were sought (e.g. for all measures, time points, analyses), and if not, the methods used to decide which results to collect.	Page 4

Section and Topic	Item #	Checklist item	Location where item is reported
	10b	List and define all other variables for which data were sought (e.g. participant and intervention characteristics, funding sources). Describe any assumptions made about any missing or unclear information.	Page 4
Study risk of bias assessment	11	Specify the methods used to assess risk of bias in the included studies, including details of the tool(s) used, how many reviewers assessed each study and whether they worked independently, and if applicable, details of automation tools used in the process.	Page 3-Page 4
Effect measures	12	Specify for each outcome the effect measure(s) (e.g. risk ratio, mean difference) used in the synthesis or presentation of results.	Page 4
Synthesis methods	13a	Describe the processes used to decide which studies were eligible for each synthesis (e.g. tabulating the study intervention characteristics and comparing against the planned groups for each synthesis (item #5)).	Page 15-Page 18
	13b	Describe any methods required to prepare the data for presentation or synthesis, such as handling of missing summary statistics, or data conversions.	--
	13c	Describe any methods used to tabulate or visually display results of individual studies and syntheses.	--
	13d	Describe any methods used to synthesize results and provide a rationale for the choice(s). If meta-analysis was performed, describe the model(s), method(s) to identify the presence and extent of statistical heterogeneity, and software package(s) used.	Page 4
	13e	Describe any methods used to explore possible causes of heterogeneity among study results (e.g. subgroup analysis, meta-regression).	Page 4
	13f	Describe any sensitivity analyses conducted to assess robustness of the synthesized results.	Page 4
Reporting bias assessment	14	Describe any methods used to assess risk of bias due to missing results in a synthesis (arising from reporting biases).	--
Certainty assessment	15	Describe any methods used to assess certainty (or confidence) in the body of evidence for an outcome.	Page 4
RESULTS			
Study selection	16a	Describe the results of the search and selection process, from the number of records identified in the search to the number of studies included in the review, ideally using a flow diagram.	Page 4
	16b	Cite studies that might appear to meet the inclusion criteria, but which were excluded, and explain why they were excluded.	Page 4

Section and Topic	Item #	Checklist item	Location where item is reported
Study characteristics	17	Cite each included study and present its characteristics.	Page 4, Page 15-Page 18
Risk of bias in studies	18	Present assessments of risk of bias for each included study.	Page 4-Page 5
Results of individual studies	19	For all outcomes, present, for each study: (a) summary statistics for each group (where appropriate) and (b) an effect estimate and its precision (e.g. confidence/credible interval), ideally using structured tables or plots.	Page 5
Results of syntheses	20a	For each synthesis, briefly summarise the characteristics and risk of bias among contributing studies.	Page 5
	20b	Present results of all statistical syntheses conducted. If meta-analysis was done, present for each the summary estimate and its precision (e.g. confidence/credible interval) and measures of statistical heterogeneity. If comparing groups, describe the direction of the effect.	Page 5-Page 6
	20c	Present results of all investigations of possible causes of heterogeneity among study results.	Page 5
	20d	Present results of all sensitivity analyses conducted to assess the robustness of the synthesized results.	Page 5
Reporting biases	21	Present assessments of risk of bias due to missing results (arising from reporting biases) for each synthesis assessed.	--
Certainty of evidence	22	Present assessments of certainty (or confidence) in the body of evidence for each outcome assessed.	Page 5-Page 6
DISCUSSION			
Discussion	23a	Provide a general interpretation of the results in the context of other evidence.	Page 6-Page 8
	23b	Discuss any limitations of the evidence included in the review.	Page 7
	23c	Discuss any limitations of the review processes used.	Page 7
	23d	Discuss implications of the results for practice, policy, and future research.	Page 7-Page 8
OTHER INFORMATION			

Section and Topic	Item #	Checklist item	Location where item is reported
Registration and protocol	24a	Provide registration information for the review, including register name and registration number, or state that the review was not registered.	Page 3
	24b	Indicate where the review protocol can be accessed, or state that a protocol was not prepared.	Page 3
	24c	Describe and explain any amendments to information provided at registration or in the protocol.	Page 3
Support	25	Describe sources of financial or non-financial support for the review, and the role of the funders or sponsors in the review.	Page 8
Competing interests	26	Declare any competing interests of review authors.	Page 8
Availability of data, code and other materials	27	Report which of the following are publicly available and where they can be found: template data collection forms; data extracted from included studies; data used for all analyses; analytic code; any other materials used in the review.	--

Supplementary Table S2 Search strategy and number of retrieved articles from different databases (*n*=6)

		Search Strategy	PubMed	Scopus	Cochrane Central	Web of Science	Embase	Epistemonikos
#1	**Condition (Co)**	("Treatment Adherence and Compliance"[Mesh] OR "Patient Acceptance of Health Care"[Mesh] OR "never treated"[tiab] OR "never treatment"[tiab] OR "never-treated"[tiab] OR "never-treatment"[tiab] OR "systematic non-compliance"[tiab] OR "systematic non-adherence"[tiab] OR "systematic noncompliance"[tiab] OR "non-treatment"[tiab] OR "non-compliance"[tiab] OR "non-adherence"[tiab] OR "noncompliance"[tiab] OR "nonadherence"[tiab] OR "unreached"[tiab] OR "untreated"[tiab] OR "missed population"[tiab] OR "missed individuals"[tiab] OR "persistent non-compliance"[tiab] OR "persistent noncompliance"[tiab] OR "treatment coverage"[tiab] OR "compliance"[tiab] OR "adherence"[tiab] OR "prevalence"[tiab] OR "determinant*"[tiab] OR "factor*"[tiab] OR "predictor*"[tiab] OR "barrier*"[tiab] OR "refusal"[tiab])	**5,50,694**	**28,807,562**	**9,77,323**	**18,032,420**	**1,96,42,961**	**4,277,382**
#2	**Context (Co)**	("Mass Drug Administration"[Mesh] OR "mass drug administration"[tiab] OR "mass drug administrations"[tiab] OR "mass treatment"[tiab] OR "preventive chemotherapy"[tiab] OR "MDA"[tiab] OR "GPELF"[tiab])	**1,08,852**	**2,32,544**	**32,282**	**1,57,820**	**2,26,050**	**73,652**

#3	**Population (Pop)**	("Elephantiasis, Filarial"[Mesh] OR "Filariasis"[Mesh] OR "Wuchereria bancrofti"[Mesh] OR "Brugia malayi"[Mesh] OR "lymphatic filariasis"[tiab] OR "filariasis"[tiab] OR "elephantiasis"[tiab] OR "bancroftian filariasis"[tiab] OR "brugian filariasis"[tiab] OR "Wuchereria bancrofti"[tiab] OR "Brugia malayi"[tiab] OR "Brugia timori"[tiab])	**21,397**	**27,526**	**7,326**	**64,705**	**72,826**	**29,356**
#4	**#1 AND #2 AND #3**	("Treatment Adherence and Compliance"[MeSH Terms] OR "Patient Acceptance of Health Care"[MeSH Terms] OR "never-treated"[Title/Abstract] OR "never-treatment"[Title/Abstract] OR "never-treated"[Title/Abstract] OR "never-treatment"[Title/Abstract] OR "systematic non-compliance"[Title/Abstract] OR "systematic non-adherence"[Title/Abstract] OR "systematic noncompliance"[Title/Abstract] OR "non-treatment"[Title/Abstract] OR "non-compliance"[Title/Abstract] OR "non-adherence"[Title/Abstract] OR "noncompliance"[Title/Abstract] OR "nonadherence"[Title/Abstract] OR "unreached"[Title/Abstract] OR "untreated"[Title/Abstract] OR "missed population"[Title/Abstract] OR "missed individuals"[Title/Abstract] OR "persistent non-compliance"[Title/Abstract] OR "persistent noncompliance"[Title/Abstract]) AND ("Mass Drug Administration"[MeSH Terms] OR "Mass Drug Administration"[Title/Abstract] OR "mass	**143**	**192**	**32**	**176**	**110**	**23**

		drug administrations"[Title/Abstract] OR "mass treatment"[Title/Abstract] OR "preventive chemotherapy"[Title/Abstract] OR "MDA"[Title/Abstract] OR "GPELF"[Title/Abstract]) AND ("elephantiasis, filarial"[MeSH Terms] OR "Filariasis"[MeSH Terms] OR "Wuchereria bancrofti"[MeSH Terms] OR "Brugia malayi"[MeSH Terms] OR "lymphatic filariasis"[Title/Abstract] OR "Filariasis"[Title/Abstract] OR "elephantiasis"[Title/Abstract] OR "bancroftian filariasis"[Title/Abstract] OR "brugian filariasis"[Title/Abstract] OR "Wuchereria bancrofti"[Title/Abstract] OR "Brugia malayi"[Title/Abstract] OR "Brugia timori"[Title/Abstract])						

Supplementary Table S3 Quality Assessment of the selected articles by AXIS Tool for Observational Studies (*n*=18)

**Questions**	Krentel et al, 2021	Manyeh et al, 2020	Boyd et al, 2010	Titaley et al, 2022	Talbot et al, 2008	Duguay et al, 2021	Dickson et al, 2021	Kisoka et al, 2014	Jabir et al, 2024	Pudasainee et al, 2025	Agarwal & Maharshi, 2024	Niles et al, 2021	El-Setouhy et al, 2007	Lau et al, 2020	Graves et al, 2020	Willis et al, 2020	Coutts et al, 2017	Fimbo et al, 2020	**Responses**		
																			**Yes No. (%)**	**No No. (%)**	**DK No. (%)**
Aims of the study clear?	Y	Y	Y	Y	Y	Y	Y	Y	Y	Y	Y	Y	Y	Y	Y	Y	Y	Y	18 (100)	--	--
Study design appropriate to stated aim?	Y	Y	Y	Y	Y	Y	Y	Y	Y	Y	Y	Y	Y	Y	Y	Y	Y	Y	18 (100)	--	--
Sample size justified?	N	N	N	Y	Y	N	N	Y	Y	Y	Y	N	Y	N	N	Y	N	Y	9 (50)	9 (50)	--
Target population clearly defined?	Y	Y	Y	Y	Y	Y	Y	Y	Y	Y	Y	Y	Y	Y	Y	Y	Y	Y	18 (100)	--	--
Sample frame represented target population?	Y	Y	Y	Y	Y	Y	Y	Y	Y	Y	Y	Y	Y	Y	Y	Y	Y	Y	18 (100)	--	--
Selection process likely to select subjects representative of target population?	Y	Y	Y	Y	Y	Y	Y	Y	Y	Y	Y	Y	Y	Y	Y	Y	Y	Y	18 (100)	--	--
Measures undertaken to address and categorise non-responders?	N	N	N	N	N	N	N	N	N	DK	N	N	N	N	Y	Y	Y	Y	4 (22.22)	13 (72.22)	1 (5.55)
Outcome variables	Y	Y	Y	Y	Y	Y	Y	Y	Y	Y	Y	Y	Y	Y	Y	Y	Y	Y	18 (100)	--	--

appropriate to aims of study?																					
Instruments trialed, piloted or published previously?	Y	Y	Y	Y	N	Y	Y	Y	Y	Y	Y	Y	Y	Y	Y	Y	Y	Y	17 (94.44)	1 (5.56)	--
Statistical significance and/or precision estimates clear?	Y	Y	Y	Y	Y	Y	Y	Y	Y	Y	Y	Y	Y	Y	Y	Y	Y	Y	18 (100)	--	--
Methods sufficiently described to be repeatable?	Y	Y	Y	Y	Y	Y	Y	Y	Y	Y	Y	Y	Y	Y	Y	Y	Y	Y	18 (100)	--	--
Basic data adequately described?	Y	Y	Y	Y	Y	Y	Y	Y	Y	Y	Y	Y	Y	Y	Y	Y	Y	Y	18 (100)	--	--
Does response rate raise concerns about non-response bias?	DK	DK	DK	DK	N	DK	N	N	N	N	DK	N	DK	DK	DK	N	N	DK	--	8 (44.45)	10 (55.55)
Information about non-responders described?	N	N	N	N	N	N	N	N	Y	DK	N	N	N	N	N	Y	Y	N	3 (16.67)	14 (77.78)	1 (5.55)
Results internally consistent?	Y	Y	Y	Y	Y	Y	Y	Y	Y	Y	Y	Y	Y	Y	Y	Y	Y	Y	18 (100)	--	--
Results presented described in methods?	Y	Y	Y	Y	Y	Y	Y	Y	Y	Y	Y	Y	Y	Y	Y	Y	Y	Y	18 (100)	--	--
Discussions and	Y	Y	Y	Y	Y	Y	Y	Y	Y	Y	Y	Y	Y	Y	Y	Y	Y	Y	18 (100)	--	--

conclusions justified by results?																					
Were the limitations of the study discussed?	Y	Y	Y	Y	Y	Y	Y	Y	Y	Y	Y	Y	Y	Y	Y	Y	Y	Y	18 (100)	--	--
Were there any funding sources or conflicts of interest that may affect the authors' interpretation of the results?	N	N	N	N	N	N	N	N	N	N	N	N	N	N	Y	N	N	N	--	18 (100)	--
Was ethical approval or consent of participants attained?	Y	Y	Y	Y	Y	Y	Y	Y	Y	Y	Y	Y	Y	Y	N	Y	Y	Y	18 (100)	--	--

DK: Don't know

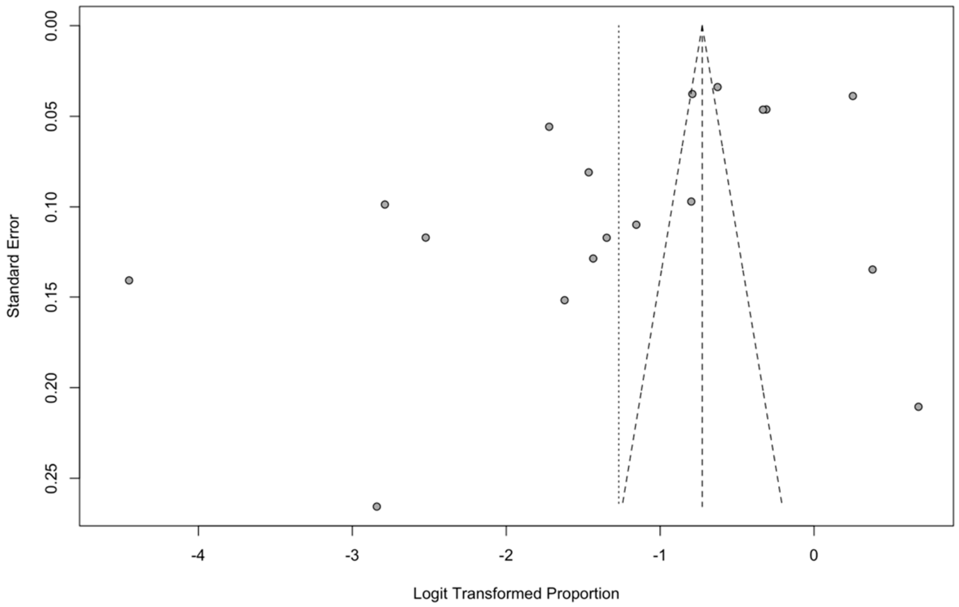

Supplementary Figure S1 Funnel plot asymmetry showing the publication bias (n=18)

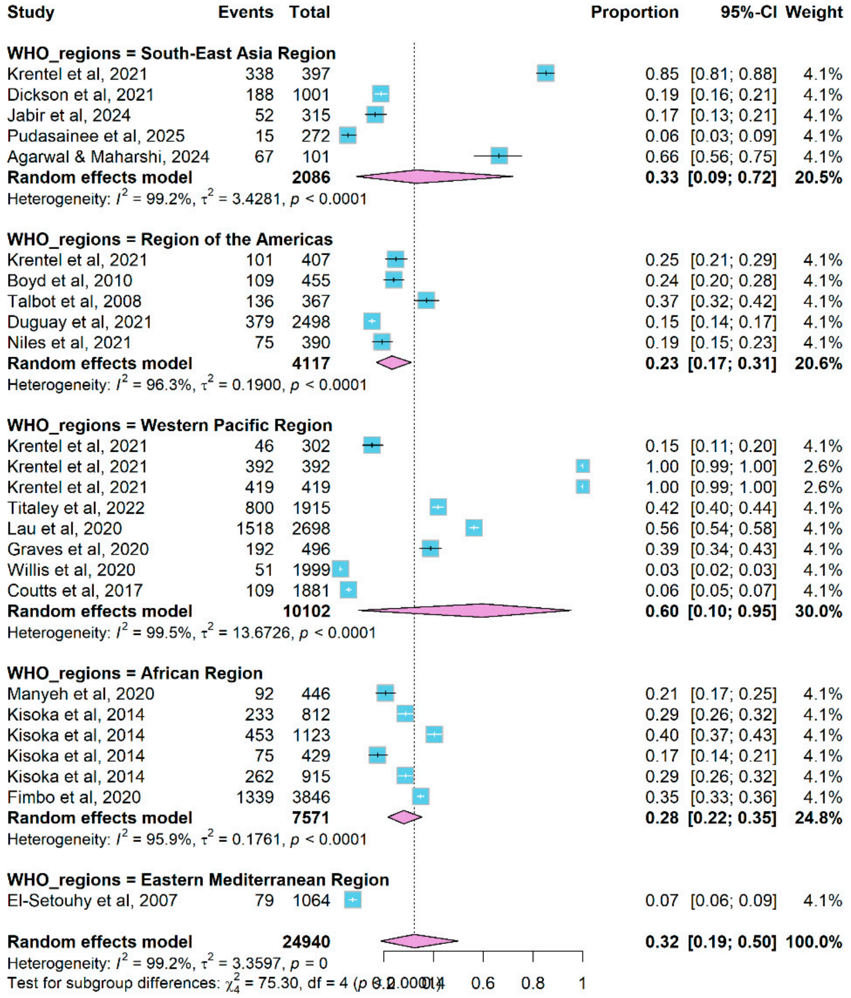

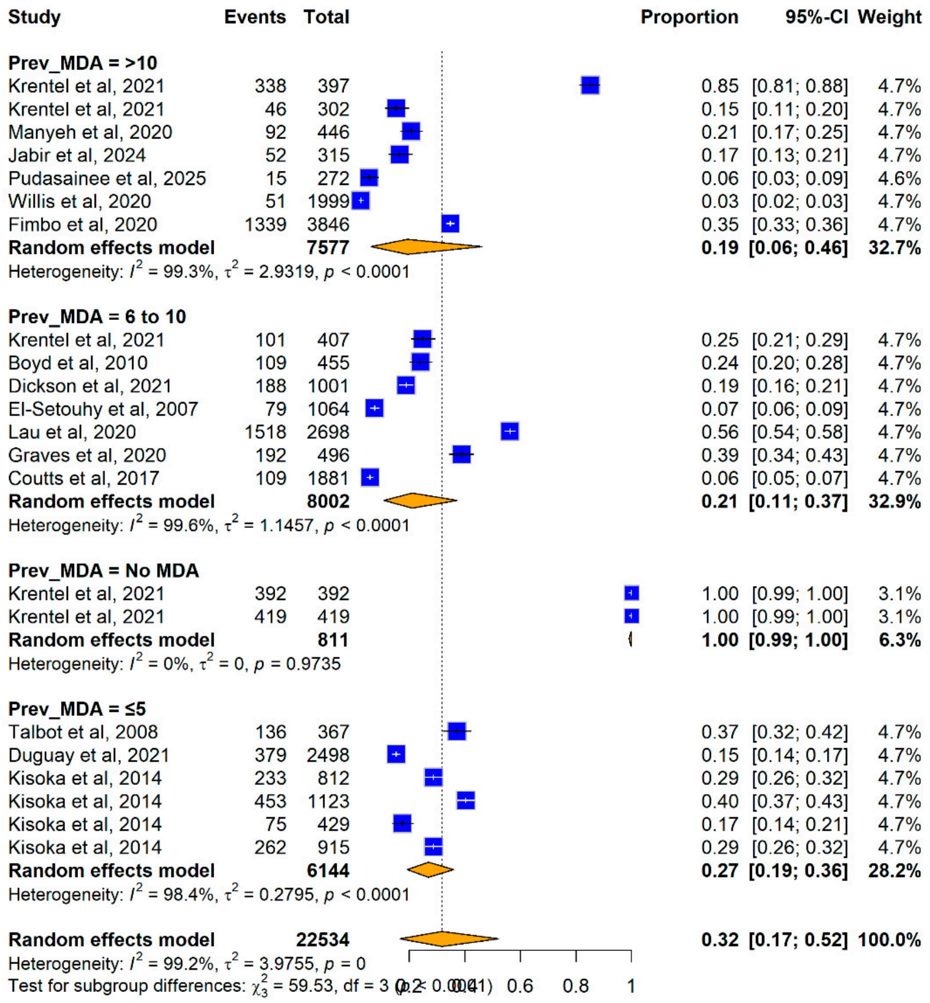

Supplementary Figure S2 Subgroup analysis showing the pooled prevalence of individuals who were never treated with MDA based on WHO regions, and the number of previous MDA rounds.

Study	Proportion	95%-CI	Tau2	Tau	I2
Omitting Krentel et al, 2021	0.21	[0.13; 0.32]	1.2250	1.1068	99.3%
Omitting Manyeh et al, 2020	0.23	[0.14; 0.36]	1.4625	1.2093	99.5%
Omitting Boyd et al, 2010	0.23	[0.14; 0.36]	1.4640	1.2099	99.5%
Omitting Titaley et al, 2022	0.22	[0.13; 0.35]	1.4169	1.1903	99.5%
Omitting Talbot et al, 2008	0.22	[0.13; 0.35]	1.4362	1.1984	99.5%
Omitting Duguay et al, 2021	0.24	[0.14; 0.37]	1.4469	1.2029	99.4%
Omitting Dickson et al, 2021	0.23	[0.14; 0.36]	1.4598	1.2082	99.5%
Omitting Kisoka et al, 2014	0.23	[0.14; 0.35]	1.4542	1.2059	99.5%
Omitting Jabir et al, 2024	0.24	[0.14; 0.37]	1.4518	1.2049	99.5%
Omitting Pudasainee et al, 2025	0.25	[0.16; 0.37]	1.2991	1.1398	99.5%
Omitting Agarwal & Maharshi, 2024	0.21	[0.13; 0.33]	1.2505	1.1182	99.5%
Omitting Niles et al, 2021	0.23	[0.14; 0.36]	1.4601	1.2083	99.5%
Omitting El-Setouhy et al, 2007	0.25	[0.15; 0.37]	1.3521	1.1628	99.5%
Omitting Lau et al, 2020	0.22	[0.13; 0.33]	1.3302	1.1533	99.4%
Omitting Graves et al, 2020	0.22	[0.13; 0.35]	1.4300	1.1958	99.5%
Omitting Willis et al, 2020	0.26	[0.17; 0.37]	1.0869	1.0426	99.4%
Omitting Coutts et al, 2017	0.25	[0.15; 0.37]	1.3026	1.1413	99.4%
Omitting Fimbo et al, 2020	0.23	[0.14; 0.35]	1.4440	1.2017	99.5%
**Random effects model**	**0.23**	**[0.14; 0.35]**	**1.3811**	**1.1752**	**99.5%**

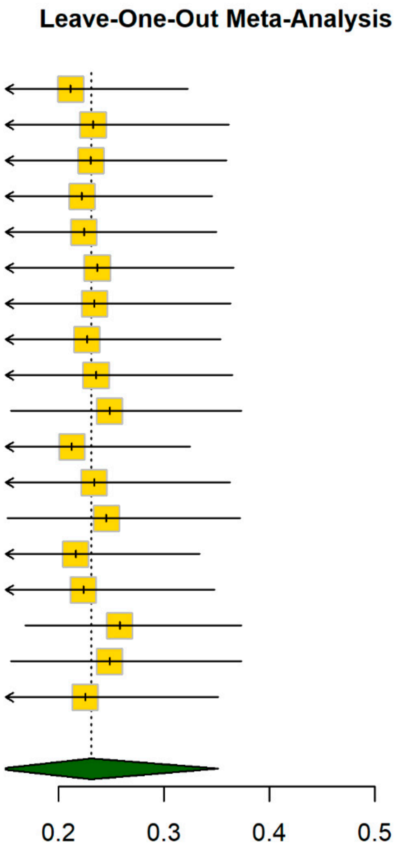

Supplementary Figure S3 Leave-one-out sensitivity analysis showing the stability of the pooled estimate

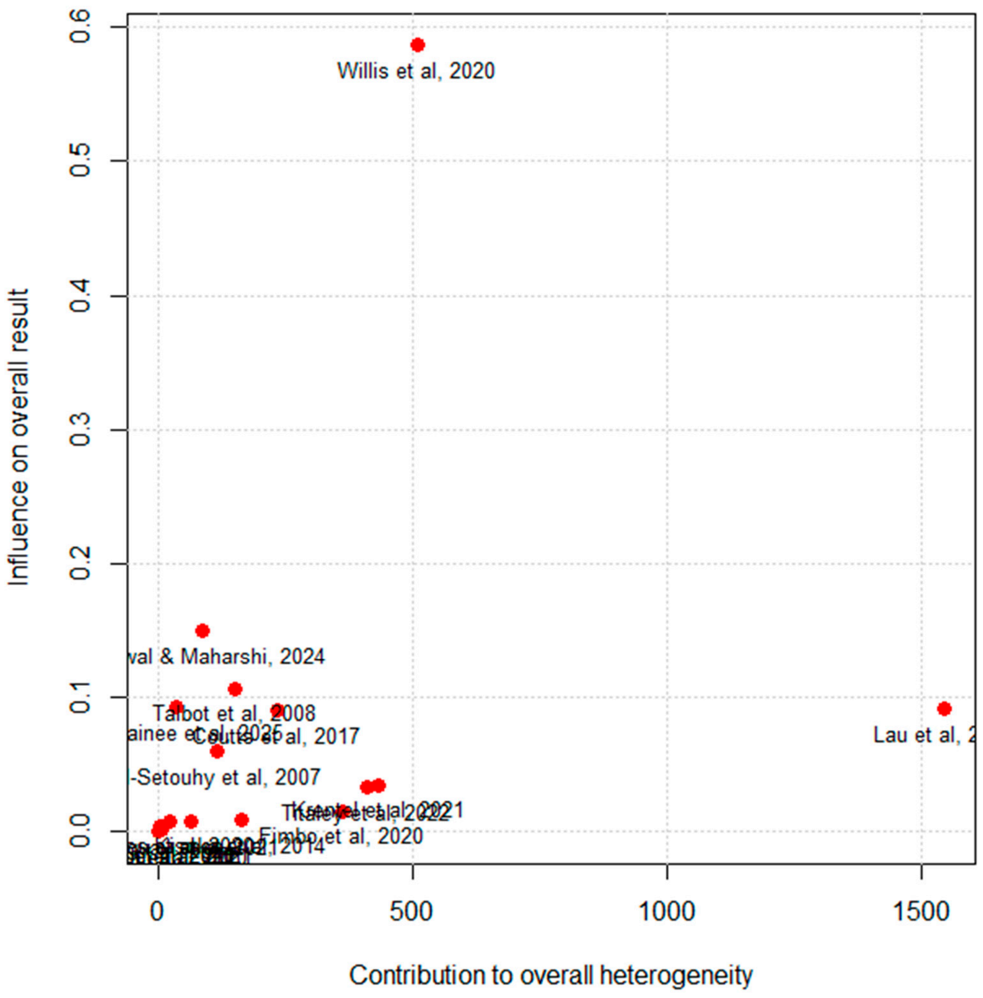

Supplementary Figure S4 Baujat Plot showing the studies that influenced the overall pooled estimates

Certainty Assessment							Summary of Findings	
**№ of participants (studies)**	**Risk of bias**	**Inconsistency**	**Indirectness**	**Imprecision**	**Other considerations**	**Certainty of evidence**	**Anticipated absolute effects (95% CI)**	**Comments**
**Prevalence of never treated individuals**								
27,225(18)	Serious^a^	Very serious^b^	Not serious	Serious^c^	None^d^	⊕◯◯◯ VERY LOW	220 per 1,000(130 to 340)	Substantial heterogeneity (I2≈99%).
**Sex differences (male vs female)**								
4,453(4)	Serious^a^	Serious^b^	Not serious	Serious^c^	None	⊕◯◯◯ VERY LOW	Males: 410 per 1,000Females: 390 per 1,000	Small difference; estimates highly heterogeneous.
**Regional variation (WHO regions)**								
25,306(17)	Serious^a^	Very serious^b^	Serious^e^	Serious^c^	None^d^	⊕◯◯◯ VERY LOW	African region: 290 per 1,000Americas: 270 per 1,000SE Asia: 210 per 1,000	Wide variability across settings.
**Association with number of MDA rounds**								
22,900(14)	Serious^a^	Serious^b^	Serious^e^	Serious^c^	None	⊕◯◯◯ VERY LOW	<6 rounds: 330 per 1,0006–10 rounds: 200 per 1,000>10 rounds: 100 per 1,000	Suggests declining trend but based on observational evidence.
**Factors associated with never treatment**								
6,348(6)	Serious^a^	Serious^b^	Serious^e^	Serious^c^	None	⊕◯◯◯ VERY LOW	Not pooled (Directional evidence)	Fear of side effects, low knowledge, social & programmatic barriers.

a. Downgraded one level for serious risk of bias due to methodological limitations in the included cross-sectional surveys (e.g., recall bias in self-reported MDA compliance).
b. Downgraded for inconsistency due to substantial statistical heterogeneity ($I^2 > 90\%$) and wide variance in point estimates across included studies.
c. Downgraded one level for imprecision due to wide confidence intervals and the event rate relative to the sample sizes across varied sub-groups.
d. In accordance with GRADE guidance for proportion meta-analyses, visual asymmetry in the funnel plot was interpreted as small-study effects and clinical/methodological heterogeneity rather than publication bias. Publication bias is assessed as undetected to avoid double-downgrading alongside inconsistency.
e. Downgraded one level for indirectness due to the broad regional pooling and varying programmatic implementations of MDA across different geographic settings.

Supplementary Figure S5 GRADE analysis was conducted based on the key quantitative outcomes and a narrative outcome
